# Antifungal Preservation of Food by Lactic Acid Bacteria

**DOI:** 10.3390/foods11030395

**Published:** 2022-01-29

**Authors:** Ahmad Nasrollahzadeh, Samira Mokhtari, Morteza Khomeiri, Per E. J. Saris

**Affiliations:** 1 Department of Food Science and Technology, Urmia University, Urmia 49171, Iran; 2 Nobonyad Nasr Food Industry Specialists Company, Tehran 11369, Iran; 3 Department of Microbiology, Faculty of Agriculture and Forestry, University of Helsinki, 00014 Helsinki, Finland; samira.mokhtari@helsinki.fi; 4 Department of Food Science and Technology, Gorgan University of Agricultural Sciences and Natural Resources, Gorgan 49189, Iran; khomeiri@gau.ac.ir

**Keywords:** synthetic preservatives, preservation enhancement, metabolites, supplementation with LAB

## Abstract

Fungal growth and consequent mycotoxin release in food and feed threatens human health, which might even, in acute cases, lead to death. Control and prevention of foodborne poisoning is a major task of public health that will be faced in the 21st century. Nowadays, consumers increasingly demand healthier and more natural food with minimal use of chemical preservatives, whose negative effects on human health are well known. Biopreservation is among the safest and most reliable methods for inhibiting fungi in food. Lactic acid bacteria (LAB) are of great interest as biological additives in food owing to their Generally Recognized as Safe (GRAS) classification and probiotic properties. LAB produce bioactive compounds such as reuterin, cyclic peptides, fatty acids, etc., with antifungal properties. This review highlights the great potential of LAB as biopreservatives by summarizing various reported antifungal activities/metabolites of LAB against fungal growth into foods. In the end, it provides profound insight into the possibilities and different factors to be considered in the application of LAB in different foods as well as enhancing their efficiency in biodetoxification and biopreservative activities.

## 1. Introduction

Fungi are among the most serious food-spoiling micro-organisms threatening the quality and health of food, food products, and feed [1]. Fungal plant-pathogens destroy up to 30% of crop products, and spoiling fungi and their toxins contaminate about 25% of raw materials produced by agriculture worldwide [2]. It is estimated that the annual economic loss caused by the spoilage of bread by fungi will reach to more than EUR 200 million in Western Europe [3].

The disadvantages of using synthetic preservatives such as the formation of carcinogenic nitrosamines in food are well known, though mold species are also becoming resistant to them [4,5]. The biopreservation of food products by natural and biological compounds may be a satisfactory alternative to solving microbial spoilage of food and food products and its consequent economic loss, which will also contribute to reducing the incidence of foodborne illnesses [6].

According to extensive studies in recent decades, LAB being able to produce active compounds such as fatty acids, organic acids, hydrogen peroxide, peptides, and reuterin represent ideal biopreservatives for conventional chemical antifungal preservatives against spoilage and toxigenic compounds in food [7,8]. A total of 25% of Europe’s diet and 60% of the diet of many developing countries is composed of fermented food, and LAB play a great role in the fermentation process [9,10]. In addition, LAB cultures isolated from native fermented food products with probiotic attributes and mycotoxin binding may be of immense value in decontaminating mycotoxins in food [11,12].

This review aimed to summarize the capability of LAB as green preservatives in different foods by highlighting their antifungal substances and mechanisms of their action. Moreover, foodborne diseases caused by pathogenic fungi as well as the hazards of synthetic preservatives for human health were outlined. Finally, a comprehensive insight into various aspects of the application of LAB as biopreservatives in foods was provided. 

## 2. Foodborne Diseases

Foodborne diseases (also called foodborne infection or food poisoning) comprise a wide spectrum of diseases resulted from the ingestion of foodstuff spoilage or pathogen microorganisms and toxic chemicals. Foodborne diseases count as a considerable cause of morbidity and mortality, which subsequently pose a remarkable impediment to socioeconomic development all around the world [13]. Since many different pathogenic microorganisms can contaminate food, there is a wide variety of foodborne infections. The Centers for Disease Control and Prevention (CDC) estimated that each year in the United States of America, 48 million people become sick as a result of foodborne illness, 128,000 people are hospitalized, and 3000 people die [14]. According to the WHO, unsafe food causes 600 million cases of foodborne diseases and 420,000 deaths annually worldwide, of which 30% belong to children under 5 years of age. The WHO estimated that eating unsafe food leads to the loss of 33 million years of lives globally each year [15]. The production and release of mycotoxins in food is the most important and dangerous effect caused by fungi to human health [16]. 

## 3. Synthetic Preservatives and Hazards of Their Use

Synthetic preservatives are substances of chemical origin that inhibit the growth of spoilage microorganisms. Some examples are benzoates, sorbates, propionate, EDTA, nitrites, and sulfites [17]. The majority of preservatives used today are synthetic rather than natural, and several of them potentially pose life-threatening side effects over time for humans as well as negative impacts on the environment [18]. Researchers have reported that synthetic preservatives can cause serious health hazards such as cancer, allergy, asthma, hyperactivity, and damage to the nervous system [19,20]. A scientific report described the cumulative behavioral effects of bread preservative on children. Daily consumption of preservative in foods has the potential to cause irritability, restlessness, inattention, and sleep disturbance in children [21]. Table 1 shows the most common synthetic antifungal preservatives, their negative effect on human health, and fungi that have developed partial resistance to them.

Benzoates mainly inhibit mold, yeasts, and bacteria in liquid environments such as acidic and soft drinks. Sodium benzoate is the most common salt of benzoate used in carbonated drinks, fruit juices, and some other foods with a pH of 3.6 or lower. It is established that benzoate can react with ascorbic acid in drinks and produce benzene, which is a carcinogen [17]. It is also reported to influence neurotransmission and cognitive functioning [33]. Although sodium benzoate is regarded as safe by major regulatory agencies, there are still questions over its adverse effects on human health. Sodium benzoate intake of above 5 mg/kg resulted in allergy and hyperactivity. Sodium benzoate has the potential to cause changes in the cell cycle and impairment in DNA as well as being considered as genotoxic and clastogenic [34]. 

Propionates inhibit mold growth in baked goods [17]. Although regarded as GRAS by FDA, there is still a lack of clarity on the metabolic effects of propionate in humans. Propionate may cause hyperinsulinemia, promoting adiposity and metabolic abnormalities over time [35]. Propionate preservatives are also reported to contribute to or cause visual irritability, restlessness, inattention, and sleep disturbance in some children [21]. 

Sorbates prevent mold/yeast growth in food products [17]. Even though sorbate is legally used in the food industry, it still has the potential to cause harmful side effects if consumed in quantities higher than the standard limits or if used long-term [36]. Various research results showed that the increased potassium sorbate intake above 25 mg/kg may lead to producing mutagenic compounds and inducing chromosome damage and DNA breakage and irritation to the respiratory epithelium [36,37]. 

Apart from negative impacts on health, synthetic preservatives may also adversely affect the organoleptic properties of the food. One of the serious problems in cheese preserved with sorbate is the decomposition of sorbic acid and potassium sorbate to trans-1, 3-pentadiene by resistant strains, and the consequent undesirable taste and odor in cheese, known as kerosene [38,39]. According to Ferrand et al. [40], sorbates might influence the taste of the food, though they are physiologically harmless and less toxic compared to benzoates [40].

Some fungi and yeasts have acquired the ability to resist chemical treatments and preservatives, which consequently creates the demand for a higher dose of the preservatives to be used. Frequent use of common antifungal agents is blamed for causing mutation in the target microorganisms and increasing their resistance [41,42]. It has been reported that some *Penicillium*, *Saccharomyces*, *Zygosaccharomyces*, *Rhizopus*, and *Yarrowia* strains can grow in the presence of potassium sorbate [4,5,43,44]. Additionally, *Z.* *bailii* and *P. roqueforti* isolates have been reported to be resistant to and even degrade benzoate, respectively [23,44]. These facts together with the demand for least processed foods and the potential hazards of synthetic preservative usage have directed the research sector for seeking alternatives for food preservation.

## 4. Lactic Acid Bacteria (LAB)

LAB homofermentatives are the species that produce lactic acid as the sole final product, while the heterofermentative ones produce lactic acid, CO_2_, and ethanol or acetate. At least half of the final product carbon is a form of lactate [45].

For centuries, LAB have been employed as bacteria performing a central role in a diversity of fermented foods involving milk, vegetables, meats, and sourdough by inducing rapid acidification of the raw material [46,47]. When it is used regularly, LAB-fermented food confers health benefits by strengthening the body in the battle with pathogenic bacterial infections [48].

LAB have also received considerable attention as probiotics over the past few years. Improving health by the biotransformation of different compounds in the gastrointestinal tract into bioavailable ones such as vitamins and short-chain fatty acids by LAB have been reported [49,50]. Immune modulation, anticarcinogenic and antitumor activity, the reduction of cholesterol, alleviation of lactose intolerance, normalization of stool transit, hepatic encephalopathy, and treatment of peptic ulcers are a number of health benefits and indicate the safety of probiotics LAB. Additionally, some modes of action of probiotic LAB are acid tolerance, adhesion to mucus and epithelial cells, production of antimicrobial compounds, and immune stimulation [51,52,53]. 

### 4.1. LAB as Green Preservatives in Food Systems

Fermentation of some foods by LAB strains with antifungal properties has been demonstrated to reduce chemical preservative usage in the food. According to Axel et al. [54], the use of sourdough fermented with specific strains of antifungal LAB can reduce chemical preservatives in bakery products [54].

LAB can be used as natural compounds to replace the chemical preservatives and are associated with health-promoting and probiotic properties [55]. LAB strains with antifungal activity also have the potential to work in synergy with synthetic preservatives. A combination of propionate and sorbate with acetic acid was shown to represent synergistic effects against fungal species of *P. roqueforti* and *A. niger* [56]. In another study, sourdough fermented by antifungal *Lactiplantibacillus* *plantarum* strains was studied for inhibition activity against *Fusarium culmorum*, *A. niger*, or *P. expansum* spores. Strong synergistic activity was reported when a combination of calcium propionate and the sourdoughs fermented by *L. plantarum* into the bread formulation was applied. The reduced use of calcium propionate up to 1000 ppm maintained inhibition only when the antifungal sourdough was added. Additionally, the increase in shelf life was interestingly higher than that obtained using calcium propionate alone (3000 ppm) [28]. 

In some research, in situ addition of LAB into food and feed was proven to delay fungal growth. Some examples are in fruits and vegetables, sour cream and semi-hard cheese, quinoa, and rice bread [54,57,58].

In situ application of LAB strains with antifungal activity in some foods have proven potential to act better than synthetic preservatives and competency to replace them in the foods. Rice dough fermented by some LAB isolated from kimchi resisted against three fungal species of *Cladosporium* sp. YS1, *Penicillium crustosum* YS2, and *Neurospora* sp. YS3 much better than that of 0.3% calcium propionate [59]. One *Leuconostoc* and five *Lactobacillus* strains surface sprayed on bakery products were shown to delay the growth of some resistant and semi-resistant fungi to calcium propionate, potassium sorbate, and sodium benzoate [26]. In the study of Mandal, Sen, and Mandal [60], the antifungal compound of *Pediococcus acidilactici* LAB 5 at a high dilution (0.43 mg mL^−1^) exerted a greater inhibition of *Curvularia lunata* conidia than sodium benzoate [60]. Valerio et al. [61] also reported that *Leuconostoc citreum*, *Weissella cibaria*, and *Lactobacillus rossiae* isolated from Italian durum wheat semolina inhibit fungal strains of *A. niger*, *P. roqueforti*, and *Endomyces fibuliger* to the same or a higher extent in comparison with calcium propionate. The results of the study indicated a potent inhibitory activity of the ten LAB strains used in their study compared to that obtained with calcium propionate (0.3% *w*/*v*) against the most widespread contaminant of bakery products, *P. roqueforti* [61].

### 4.2. Antifungal Activity Spectrum of LAB

LAB have a reported potential use as adjunct or starter cultures to inhibit fungi growth in the final products such as fruit and vegetables, dairy, and bakery. Twenty LAB isolates from fermented cassava were investigated against fungal pathogens associated with the spoilage of vegetables and fresh fruits. Strong inhibition of the radial growth and spores of the fungal pathogens was observed when the products were inoculated with the antifungal metabolites produced by the strains [57].

In cheese, *Lactobacillus amylovorus* DSM 19280 was used as an adjunct culture in a cheddar cheese model system contaminated with *P. expansum* spores. The presence of the strain resulted in a four-day delay in *Penicillium* growth on the cheddar cheese compared with the control [62]. *Lactobacillus rhamnosus* A238 was also shown alone or in combination with *Bifidobacterium animalis* to inhibit mold growth on cottage cheese for at least 21 days at 6 °C [63]. In another study, 12 selected *L. plantarum* isolates were inoculated into cottage cheese challenged with *Penicillium commune*. All the isolates were found to prevent the obvious *P. commune* growth on cottage cheese by between 14 and more than 25 days longer than the control [64]. *Lactobacillus brevis* and *Enterococcus faecium* isolated from “chal”, a product from yogurt, reduced the growth of *Rhodotorula glutinis* in doogh, diluted yogurt, over 15 days of storage [65]. 

In sour cream and semi-hard cheeses, Lactobacillus paracasei CIRM-BIA1759 and L. rhamnosus CIRM-BIA1761 were tested as adjunct cultures. In situ assays showed that the strains postponed the growth of *P. commune*, *Rhodotorula mucilaginosa*, and *Mucor racemosus* on sour cream for 2–24 days and also delayed the growth of *P. commune* in semi-hard cheese for 1–6 days [58]. Ouiddir et al. [66] tested the antifungal activity of *L. plantarum* CH1, *L. paracasei* B20, and *Leuconostoc mesenteroides* L1 in sour cream and sourdough bread challenged with fungal spoilers. The strains delayed the growth of the *Aspergillus tubingensis*, *A. flavus*, *P. commune*, and *M. racemosus* for up to 5 days in sourdough bread. In sour cream, *L*. *plantarum* CH1 and *L. paracasei* B20 completely inhibited *P. commune* growth for 5 and 3 days, respectively [66]. 

In bakery products, in situ sprays of one *Leuconostoc* and five *Lactobacillus* strains delayed one or several fungal species growths. The incorporation of the same strains in milk-bread-roll preparation also delayed fungal growths [26]. In another study, two strains of *Lactobacillus* were used for sourdough fermentation of quinoa and rice flour. *L. reuteri* R29 and *L. brevis* R2Δ fermented sourdough bread reached a shelf life of quinoa and rice from 2 to 4 days, respectively [54]. A Chinese steamed bread manufactured with *L. plantarum* CCFM259 did not show any fungal contamination until 7 days of storage, a similar level of inhibition compared with that obtained by 0.25% (*w*/*w*) calcium propionate [67]. Fermenting rice dough with some LAB isolated from kimchi greatly retarded the growth of three fungal species from *Cladosporium*, *Neurospora*, and *Penicillium* genus in the rice cakes [59].

Different LAB isolates have the potential to synergically inhibit fungal growth in food. Seven strains of LAB were selected and tested for their anti-penicillium activity to prevent *Penicillium* *chrysogenum* growth in cottage cheese. They found that some of the strains act in synergy, and their combination has potential for use as bio-preservatives in fresh cheese [63]. 

Antifungal activity of LAB depends on the pH, temperature, growth media, incubation time, nutrients, antifungal compounds, production levels, and mode of action [68]. Mandal, Sen, and Mandal [60] observed that the production of antifungal compound(s) from *P. acidilactici* LAB 5 against pathogenic fungi showed a great dependency on media specifications. TGE, and TGE + Tween 80 media did not support the production of any antifungal compounds, while the fungal growth was completely restricted in MRS agar media [60]. Another study reported that supplementation of WFH media with 2.5% olive oil and 150 mM glycerol raised the antifungal activity of *L. brevis* Lu35 and *L. reuteri* 5529, respectively [26]. The addition of linoleic acid supported the antifungal activity of *Lactobacillus hammesii* [69]. Rouse et al. [70] reported that when grown in different carbon sources, the antifungal activity of the LAB strains tested was stable, although the quantity of metabolites produced varied depending on the carbon source. Among the sugars tested, for three out of four strains, glucose and lactose were the best and worst, respectively [70].

The incubation time has been observed to greatly influence the antifungal activity of LAB. Rouse et al. [70] observed that the four tested LAB cultures were unable to grow at 10 and 42 °C, and consequently, no growth was observed. Incubation between 21 and 37 °C, however, improved growth, and the bacteria presented different levels of antifungal activity with the optimal production of the antifungal compounds between 25 and 30 °C [70]. The antifungal activity of *Lactobacillus coryniformis* subsp. *coryniformis* strain Si3 was slightly better at 30 °C as compared to 25 °C for 40 h. The production of antifungal compounds by the strain was reported to begin in the log phase and reach a maximum level in the early stages of the stationary phase followed by a drop in activity [71].

The antifungal activity of LAB has also been found to be influenced by pH. The antifungal activity of *L. plantarum* K35 was reported to be pH-dependent and favorable to acidic conditions [72]. Antifungal properties of *L. coryniformis* subsp. *coryniformis* strain Si3 were also observed at maximum at pH values of between 3.0 and 4.5, with a decrease in pH between 4.5 and 6.0, and loss at higher pH values. Readjustment of the pH to 3.6 fully returned the activity [71]. In another study, antifungal attributes of the four LAB strains were found to be good at pH 3, moderate at pH 5, and low at pH 7, although poor fungal inhibition was maintained at pH 8 [73]. The effect of temperature and pH on the antifungal properties of the *L. plantarum* strain against *Aspergillus* *fumigatus* and *Rhizopus stolonifer* in temperatures ranging from 20 °C to 40 °C and pH ranging from 4.0 to 7.0 for 48 h of incubation was investigated. A combination of 30 °C and pH 6.5 °C presented optimum antifungal activity [74].

The role of the concentration of supernatant in the antifungal activity of LAB were also highlighted by Shehata et al. (2019), where they observed that increasing the supernatant concentration of *Lactobacillus* sp. RM1 decreased the growth of *Aspergillus* *parasiticus*, *A. flavus*, and *Aspergillus carbonarius* [18]. 

Fermentation time was also found to be effective in the antifungal activity of LAB. Longer fermentation times of barley malt substrate fermented by LAB resulted in higher carboxylic acids released by them against *F. culmorum* macroconidia. The maximal concentrations of the acids were obtained after 48 h of fermentation [75]. Among the four LAB strains studied by Muhialdin, Hassan, and Saari [73], the highest antifungal activity of *Lc. mesenteroides* and three *L. plantarum* occurred in different incubation times of 24 h and 48 h, respectively. They highlighted the significance of incubation time, growth stages, and temperature for the production of antifungal compounds. According to them, maximizing the production of inhibitory compounds could be obtained by determining the optimum growth conditions [73].

The inhibitory activities of LAB are strain specific. Selecting the best strain/combination of strains of LAB for biopreservation that would cause the minimum unfavorable changes in the product requires prior experiments. In a study, more than 200 yeast and 200 LAB strains were tested as biopreservatives against fungal growth during the cocoa fermentation process. The most promising candidates among all belonged to only four species of *Lactobacillus fermentum*, *L. plantarum*, *Saccharomyces cerevisiae*, and *Candida ethanolica* [76]. *Lactobacillus coryniformis* subsp. *coryniformis* strain Si3 was observed to have strong inhibitory activity against *A. fumigatus*, *P. Roqueforti*, *Aspergillus nidulans*, *Mucor hiemalis*, *Fusarium. graminearum*, *Talaromyces flavus*, *Fusarium poae*, *F. culmorum*, and *Fusarium sporotrichoides*. A weaker activity from the same strain was observed against *Kluyveromyces marxianus*, *Debaryomyces hansenii*, and *S. cerevisiae* while displaying no activity against *Sporobolomyces roseus*, *R. glutinis*, and *Pichia anomala* [71]. Further support for this is another study where the inhibitory percentage of *L. brevis* was stronger than *E. faecium* against *P. chrysogenum* [77]. 

The selected strains must be adapted or adaptable to the environmental conditions of target food, as well as the production process through which the food is prepared, so that their activity and release of antifungal compounds can be reasonably expected during storage. *L. reuteri* was added to a fermented milk product to inhibit pathogens and spoilage microorganisms. No change in the pH, acidity, soluble solids, color, or rheological aspects of the fermented milk product in the presence of reuterin was observed [78]. Fermentation of an oat-based beverage by *L. plantarum* UFG 121 also best preserved it against *F. culmorum*, causing no differences in terms of some qualitative features as compared to the control [79].

Bacterial metabolite profiles of LAB could sometimes be beneficially modulated, altering their spectrum of antifungal activity. Both the culture medium and the target fungal species determine the antifungal activity of LAB. The quality and quantity of the antifungal metabolites of *Lactobacillus pentosus* ŁOCK 0979 were reported to be dependent on the culture medium compounds. The presence of galactosyl-polyols and gal-erythritol improved the anticandidal properties of *L. pentosus* ŁOCK 0979. The addition of the culture medium of the strain conferred an inhibitory attribute against *Aspergillus brassicicola* and *A.* *niger.*

### 4.3. Antifungal Metabolites of LAB

LAB inhibitory compounds are secondary metabolites produced after 48 h of fermentation [70]. Shehata et al. [18] observed that the production of the antifungal metabolites of the LAB strain *Lactobacillus* sp. RM1 was initiated at the growth log phase (12–14 h), reached the highest at the strain stationary phase (24 h), and remained stable. The proposed mechanisms explaining the fungal inhibitory effect of LAB are competition over the available nutrients and space, clogging the pathogen’s path through the matrix, and manipulation of the spore membrane, causing viscosity and permeability [80]. Sangmanee and Hongpattarakere [72] revealed that the mechanism of antifungal action of *L. plantarum* K35 supernatant causes damage to the cytoplasmic membrane and cell wall and consequent leakage of cytoplasmic content, the formation of membrane-bound vesicles followed by the destruction of mitochondria and nuclei [72].

Lactic acid, formic acid, acetic acid, caproic acid, and phenyllactic acid (PLA), as organic acids, as well as other metabolites from LAB such as carbon dioxide, hydroxyl fatty acids, hydrogen peroxide, diacetyl, ethanol, reuterin, cyclic dipeptides, protein compounds, reutericyclin, proteinaceous, acetoin, and volatile compounds such as diacetyl are natural antimicrobial and antifungal metabolites produced by LAB [7,58]. Table 2 summarizes the number of LAB studied for their antifungal metabolites, fungal spectrum of activity, and their in situ application in the last 10 years.

The antifungal metabolites of LAB have the potential to act in synergy. The synergistic effect between the decrease in pH resulted from the production of organic acids and other antifungal metabolites of LAB poses a more efficient final antifungal activity [7]. Peyer et al. [75] demonstrated that there are synergistic effects between organic acids and phenolic acids released by some LAB strains as antifungal metabolites against *F. culmorum*. According to Alex et al. [54], the great synergistic effect between organic acids and antifungal peptides produced by LAB allow the final biopreservation attribute to be influential in bakery products [54].

Antifungal metabolites of LAB have also shown synergy with compounds of other organisms. Ruggirello et al. [76] tested some yeast and LAB strains against six spoilage fungi belonging to *Aspergillus* and *Penicillium* genera during the cocoa fermentation process. The antifungal activity was explained by the synergic production of organic acids (from the LAB) and proteinaceous compounds (from yeasts) [76]. 

The understanding of the synergy mechanism between antifungal compounds could provide insight in maximizing the impact whilst altering the involved compositions of bacteria or nutrients, eventually leading to actual application in food [75]. 

#### 4.3.1. Organic Acids

The production of organic acids is believed to determine an LAB strain’s mycotoxigenic fungi inhibition properties; the type and quantity of the acids differ from strain to strain [87]. These acids are mainly produced by LAB as a byproduct of acidification process rather than as active synthesis of metabolic compounds aimed at restricting fungi [88].

As the main acid produced by LAB, lactic acid (2-hydroxy propionic acid) is an organic acid widely distributed in nature in two forms of L and D; L lactic acid was recognized as a safe preservative by the FDA [89]. Russo et al. [79] tested the activity of some LAB strains against *Aspergillus*, *Fusarium*, and *Penicillium* and reported that lactic acid was produced at a high concentration during the growth phase as the main metabolic antifungal associated with the low pH. Lactic acid was also identified as the main antifungal compound from *E. faecium*, *L. rhamnosus*, and *L. plantarum* [90]. In the study of Baek et al. [59], the fermentation of rice dough with some LAB isolates from kimchi greatly delayed the growth of three fungal species in rice cakes. They found lactic acid and acetic acid as the main antifungal substances [59]. 

The best characterized and most important antimicrobials produced by LAB are lactic acid and acetic acid, which are bioactive in the protonated form at low pH [91]. LAB can produce a variety of compounds at low concentrations and below their minimum inhibitory concentration, which are likely to act synergistically with lactic acid and acetic acid [92,93]. Acetic acid and lactic acid were also proved to display a synergistic antifungal activity in combination; however, due to higher pKa that causes a higher level of dissociation inside the cell, acetic acid has a stronger antifungal activity [94,95]. Loubiere et al. [96] suggested that lactic acid has an inhibitory effect on the metabolism and cell proliferation, which is probably due to the synergistic effect with some of the other side fermentation products such as acetic acid and formic acid [96].

Organic acids of lactic acid, oleic acid, linoleic acid, palmitic acid, 3-PLA, stearic acid, pyroglutamic acid, and 5-oxo-2-pyrrolidine-carboxylic acid were detected as antifungal compounds from *L. plantarum* K35 inhibiting the growth and aflatoxin production of *A. flavus* and *A. parasiticus* [72]. Some other carboxylic acids including benzoic, vanillic, azelaic acid, hydrocinnamic acid, and hydroxy benzoic acid were isolated as antifungal compounds from mediums of *Weissella cibaria* PS2 and three *Lactobacillus* species [97]. Hydrocinnamic acid, azelaic acid, vanillic, p-couramic, and 4-hydroxy benzoic acid were also reported from *L. reuteri* eep1 with antifungal activity [98].

The mechanism of inhibitory activity of organic acids in the growth and activity of many pathogenic and putrefactive bacteria and fungi is attributed to creating an acidic environment and reducing the pH to below the metabolic inhibition and growth range [94]. Loubiere et al. [96] suggested that the inhibitory effect of lactic acid on the metabolism and cell proliferation is probably due to the increase in osmotic pressure of the medium. Organic acids alter plasma membrane permeability and electrochemical, killing the microorganism [80]. In other words, organic acids diffuse to the fungi through the membrane and degrade the cells, thereby releasing hydrogen ions and causing a decrease in pH [99].

For organic acids to penetrate the cell wall, they must be turned to an undissociated form. The pKa of lactic, acetic, caproic acid, and 3-phenyl-L-lactic is 3.8, 4.7, 4.9, and 3.5, respectively [99]. Therefore, in an acidic environment, they will act more efficiently in inhibiting fungi. This fact was proven in the study of Cortés-Zavaleta et al. [7], where they tested the fungal inhibition of the cell-free supernatant of 13 LAB strains against four food-spoilage fungi. The results demonstrated that the inhibition properties dropped when the pH was raised to 6.5 [7].

The production of organic acids confers extra inhibition properties to LAB by activating other antifungal compounds triggered as a result of lowering the pH [99]. Furthermore, organic acids often work in synergy with other compounds, which adds to the complexity of LAB antifungal activity [54]. There is also a synergistic effect between several organic acids together produced by LAB as antifungal compounds. The antifungal activity of *L. plantarum* CCFM259 was assessed against *P. roqueforti*. Acetic acid and PLA showed better antifungal activity than other compounds, and their mixture displayed a synergistic effect [67]. The synergistic contribution of acetic acid was reported in the extension of sourdough fermented by two *Lactobacillus* strains [54].

#### 4.3.2. Phenyllactic Acid (PLA)

PLA (2-hydroxy-3-phenyl propionic acid) is another well-studied organic acid with natural antibacterial properties derived from phenylalanine catabolism. PLA possesses a similar metabolic pathway as lactic acid and is metabolized during fermentation by the glycolytic enzyme and lactate dehydrogenase [100].

The composition of the culture medium was reported to play a great role in the quantity of PLA produced by LAB. The addition of 1.5% (*w*/*v*) phenylalanine to MRS medium of *L. reuteri* R29 significantly increased the production of PLA and, consequently, antifungal performance against *F. culmorum* [101]. The fungal inhibitory strength of PLA produced by LAB are well-established. Lavermicocca et al. [102] reported that a 10-fold-concentrated culture supernatant of *L. plantarum* 21B inhibited *Eurotium*, *Fusarium*, *Penicillium*, *A. monilia*, and *Endomyces*. Under the same conditions, 3 mg mL^−1^ calcium propionate was not effective, while sodium benzoate performed similar to *L. plantarum* 21B. The antifungal activity of *L. plantarum* 21B was attributed to PLA and 4 OHPLA isolated from the supernatant of the bacteria [102]. In a similar study, *L. plantarum* UM55 was found to produce lactic acid, PLA, OHPLA, and indole lactic acid (ILA). The acids were individually tested against *A. flavus*, and among them, PLA showed the strongest effects with the obtained IC90 for the growth inhibition of 11.9 mg mL^−1^ [103]. PLA and 3,5-Di-O-caf- feoylquinic acids were identified as the predominant antifungal compounds in cell-free supernatant of seven LAB isolated from traditional fermented Andean products with inhibitory activity against a few spoiler fungi from *Penicillium* and *Aspergillus* genus [104]. The antifungal compounds of *L. plantarum* against *A. fumigatus* and *R. stolonifera* resembled the structure of 3PLA with the formed ligands [74].

Although promoting the metabolic pathway of PLA is likely to increase the efficiency, the antifungal properties of PLA depend on a synergistic mechanism with other metabolites [101]. Cortés-Zavaleta et al. [7] tested the fungal inhibition of the cell-free supernatant of 13 LAB strains against four food-spoilage fungi. With two exceptions, all other LAB strains produced PLA ranging from 0.021 to 0.275 mM. They concluded that even if PLA cannot be the only inhibitory compound, it very likely performs in synergy with other acidic compounds from LAB [7]. PLA and acetic acid produced from *L. plantarum* CCFM259 exhibited a synergistic inhibitory effect against *P. roqueforti* [67]. A weak synergistic inhibitory effect of PLA was also reported in combination with cyclo (L-Phe–L-Pro) produced from *L. plantarum* against *A. fumigatus* and *P. roqueforti* [105]. Acetic acid, lactic acid, and PLA produced by *L. plantarum* VE56 and *Weissella paramesenteroides* LC11 exhibited synergism against *A. tubingensis*, *A. niger*, *Candida albicans*, and *P. crustosum* [106]. 

#### 4.3.3. Reuterin

Reuterin (β-hydroxy propionaldehyde) is a low-molecular-weight multi-compound system consisting of 3-HPA hydrate, 3-hydroxypropionaldehyde (3-HPA), 3-HPA dimer, and acrolein produced by the conversion of glycerol [107]. Reuterin is secreted mainly by *L. reuteri*, though some other bacterial species and genera could also secrete it [107]. Reuterin has a wide spectrum of antimicrobial properties against a range of Gram-positive and Gram-negative bacteria, bacterial spores, molds, yeasts, and protozoa [84,94]. 

The growth conditions and culture medium can alter the content of reuterin produced by LAB. Schaefer et al. [108] reported the optimum conditions for reuterin production from *L. reuteri* 1063 as culturing the cells for 16 h followed by suspension in 5 mL of 250 mM glycerol in distilled water and incubated for 2 h at 37 °C under anaerobic conditions. Another study reported that supplementation with 150 mM glycerol increased the antifungal activity of *L. reuteri* 5529 cultured in WFH medium. The enhanced antifungal activity of *L. reuteri* 5529 was linked to the production of reuterin. In a similar study, glycerol addition to the culture medium of *L. coryniformis* improved reuterin synthesis, consequently having an antifungal effect against yeast cells and fungal spores and conferring inhibition performance against a couple of new fungal strains [109].

The activity of LAB against fungi is mostly limited to antifungal rather than fungicidal. Reuterin, however, apart from antifungal activity, has also presented a fungicidal effect. Purified Reuterin produced by *L. reuteri* ATCC 53608 fungicidal activity by killing 99.9% of the indicator microorganisms at concentrations equal or below 15.6 mM. As an antifungal agent, it was then added to yogurt. In yogurt also, reuterin exhibited an antifungal effect at a concentration of 1.38 mM while a fungicidal effect at 6.9 mM [84].

The mechanism of action of reuterin has been reported to cause oxidative stress to fungal cells. Reuterin exposure *E. coli* increased the expression of genes regulated and expressed in response to periods of oxidative stress. It was determined that the aldehyde group of reuterin binds to thiol groups of small peptides and other molecules, leading to oxidative stress, which is hypothesized as the mechanism of inhibition [108]. Another proposed inhibition mechanism of reuterin is through the suppression of ribonuclease activity, which is the main enzyme mediating the biosynthesis of DNA [110], as cited in [68]. More recently, acrolein was reported as the main component conferring antimicrobial activity to reuterin [107]. 

Reuterin could be a potentially promising candidate as a food biopreservative since in vitro studies using human liver microsomes demonstrated that reuterin does not present the possibility of displaying drug interactions [63]. *P. expansum* was inhibited at concentrations of above 10 mM reuterin produced by *L. reuteri* ATCC 53608 [78]. The addition of *L. reuteri* INIA P572 with glycerol to semi-hard ewe milk cheese resulted in a lower level of 2-heptanone in cheese, which was attributed to the activity of reuterin in mold inhibition [111]. Reuterin was also found to be responsible for the antifungal performance of three *Lactobacillus* and one *Leuconostoc* strains applied in pound cake and milk bread rolls [26].

#### 4.3.4. Peptides and Cyclic Peptides

The antimicrobial peptides are chains of 5–100 amino acid attached through peptide bonds with natural origin (held together through peptide bonds [112]. Protease enzyme treatment is usually employed to determine the peptide nature of the active compounds. The treatment of *Lactobacillus fermentum* CRL 251 supernatant with trypsin, proteinase K, and pepsin decreased the antifungal activity by 50, 4, and 3%, respectively. Further ultrafiltration analysis attributed the activity to smaller fraction of peptides (<10 kDa) [113]. In the study of Magnusson and Schnürer [71], *L. coryniformis* subsp. *coryniformis* presented a strong inhibitory activity against a number of fungi and yeast strains. The activity was attributed to the production of small (3 kDa) and heat-stable proteinaceous antifungal compounds demonstrated by the alteration of activity through the treatment with proteinase K, trypsin, and pepsin [71]. A group of peptides was purified and identified from cell-free supernatants of *L. plantarum* exhibiting inhibitory activity against *A. parasiticus* and *P. expansum* by 58% and 73%, respectively [112]. A total of 37 peptides were identified in the fraction of cell-free supernatant of *L. plantarum* TE10. Treatment of bread with the fraction resulted in slight growth and a fourfold reduction in spore formation of *A. flavus* [73]. 

Smaller peptides usually possess stronger antifungal activity. Low-molecular-weight peptides (<10 KDa) isolated from the supernatants of four LAB strains represented higher antifungal inhibition against six fungi in comparison with the control supernatant [73]. 

The mechanism of action of peptides is through binding to lipid bilayers in carpet-like and puncturing channels in it, which impairs the function. They also act through peptide-lipid interaction resulting in phase separation as well as solubilizing the membrane [114].

Low-molecular-weight peptides with high heat-stability from LAB have high potential for replacing chemical preservatives commonly used in the bakery [73]. In the study of Muhialdin, Hassan, and Saari [73], they simulated the maximum heat process of food in manufacturing (121 °C for 60 min) and exposed the supernatants of four LAB strains with antifungal activity to it. The nature of the bioactive substances secreted by bacteria was found to determine whether the activity is heat sensitive and to what extent [73].

Cyclic peptides are composed of polypeptide chains linked covalently in a circular manner. The circular structure is formed either by binding either ends of the peptide chain through an amide bond, or by lactone, thioether, ether, or disulfide bonds [115]. The cyclic dipeptide properties as antifungal agents produced by LAB have been shown in several studies and reviews. *L. plantarum* CM8, *Weissella confusa* I5, *P. pentosaceus* R47, and *W. cibaria* R16 presented inhibitory activity against *P. notatum*. Concentrated supernatants were heated to 80 °C for 1 h followed by an autoclavation step (121 °C for 15 min). No significant influence of heat was observed in the activity of the supernatants. Protease sensitivity properties of the activity implied that the bioactive substances most likely have a proteinaceous nature, perhaps (cyclic) peptides [70].

Cyclo peptide (glycyl-L-leucyl), as a compound that delays the growth of fungi *Fusarium avenaceum*, was isolated from *L. plantarum* [116]. Magnusson [109] also reported the secretion of cyclic dipeptides by *P. pentosaceus* (MiLAB 024), *L. plantarum* (MiLAB 006), and *Lactobacillus sakei* (MiLAB 091). The growth condition of the LAB strain was found to be influential in the quantity of the cyclic peptides released by them. The results obtained by Ryan et al. [88] revealed that acidification of dough fermented by *L. plantarum* FST significantly increased the quantity of cis-cyclo (LPhe-L-Pro) and cis-cyclo (L-Leu-L-Pro) as compared to nonacidified dough [88]. 

The mechanism of action of antimicrobial cyclic peptides is mainly attributed to the disruption of structural integrity. They target the cell envelope components, causing lysis of the membrane or inhibiting the membrane and/or cell wall biosynthesis [117]. 

#### 4.3.5. Fatty Acids

Fatty acids are organic acids that possess a carboxyl group (-COOH) and a methyl group (-CH3) at either end [118]. Strong antifungal activities have been reported from fatty acids. Sjogren et al. [119] characterized 3-hydroxydodecanoic acid, 3-hydroxydecanoic acid, 3-3-hydroxy-5-cis-dodecenoic acid, and hydroxytetradecanoic acid from the supernatant of *L. plantarum* MiLAB 14. The hydroxy fatty acids displayed inhibition in the range 10 to >100 µg/mL and were reported to be much more effective than cyclic dipeptides against several molds and yeasts [119]. 

Fatty acids have been reported in a number of studies to be the main antifungal metabolite preserving foods fermented by LAB. Fermentation of sourdough bread and sour cream by three isolates of LAB of *L. plantarum* CH1, *L. paracasei* B20, and *Lc. mesenteroides* L1 delayed fungal growth in the final food. The main produced compounds were detected to be DL-hydroxyphenyl, 3, 3-(4-hydroxyphenyl) propionic, 4-dihydroxyhydrocinnamic, and 3-(4-hydroxy-3-methoxyphenyl) propanoic acids [66]. Black et al. [69] reported that *L. hammesii* converts linoleic acid to antifungal C_18:1_ monohydroxy fatty acids. Further supplementation of linoleic acid strengthened the antifungal activity of *L. hammesii*. Hydroxylated fatty acids synthesized by the strain were found to be responsible for the extended shelf life of sourdough fermented with *L. hammesii* and the inhibition of *A. niger* and *P. roqueforti* in the bread prepared by that [69].

Little knowledge of the antifungal mechanisms of fatty acids is available so far; however, some pathways have been proposed. Detergent-like properties of the fatty acids affect the structure of cell membranes of the cells, leading to death [119]. Antifungal fatty acids disintegrate lipid bilayers of the membranes and, consequently, cause destruction of the membrane integrity, leading to the disintegration of cells and release of intracellular proteins and electrolytes [120]. Other targets of fatty acids include protein synthesis, which may be inhibited by myristic acid analogues, fatty acid metabolism, as well as topoisomerase activity, which may be inhibited by, amongst others, acetylenic fatty acids [121].

The antifungal activity of fatty acid highly depends on the structure. In the study of Black et al. [69], unsaturated monohydroxy fatty acids were antifungally active; saturated hydroxy fatty acids and unsaturated fatty acids of oleic and stearic acids, however, did not exhibit any activity. This implies the fact that for the fatty acid to function as an antifungal agent, at least one double bond as well as one hydroxyl group along a C18 aliphatic chain should be present in the structure [69].

Pathogenic fungi are less likely to become resistant to antifungal fatty acids [121]. Other antifungal compounds targeting the membrane of fungi are more susceptible to pathogen resistance, which shortens their lifespans. However, as these substances could present synergism with antifungal fatty acids, they could alternatively provide prolonged usage, reducing the required quantity of the antifungal substances [120]. An example of the synergism of fatty acids with other compounds was provided by the study of Ndagano et al. (2011). They observed that 3-hydroxylated produced by *L. plantarum* VE56 and *W. paramesenteroides* LC11 acts in synergy with other bacterial compounds secreted by the bacteria inhibiting *A. niger*, *A. tubingensis*, *C. albicans*, and *P. crustosum* [106].

## 5. Conclusions

Fungal growth and consequent mycotoxin release in food and feed threaten human health, which might even, in acute cases, lead to death. Addressing the consumer health concern as well as the potential negative risk of using synthetic preservatives, the substitution of LAB as a green preservative could be an alternative due to their safety, health-giving benefits, and preservation properties. LAB release antifungal metabolites against fungal species, which in many cases work in strong synergy.

The application of LAB species with antifungal properties in food can reduce the occurrence of fungal spoilage and toxicity, consequently improving its shelf life as well as causing a reduction in mycotoxins. However, case investigation is required to be carried out individually for each food candidate since the presence of LAB in food can exclusively affect its physiochemical and organoleptic properties, which may or not be desirable. On the other hand, the major population of fungi contaminating a particular food should be regarded in selecting the best LAB/combinations of LAB planned for inhibiting fungal growth in the food. The reason for that is the fact that the antifungal properties of LAB are fungal strain-specific, meaning that an LAB strain might be strongly active against a fungal strain while not causing much disturbance in the viability of another strain.

Almost all antifungal metabolites of LAB present synergy with at least one other component. This fact counts as an advantage in employing LAB as antifungal bacteria in a way to group main producers of synergic components together, thereby maximizing the final activity. The composition of the medium has also been demonstrated to be a significant factor stimulating/raising the release of antifungal compounds by LAB. Therefore, the food nutrient composition is another item to take into account when selecting LAB strains to inhibit fungal growth/mycotoxin control in food. If the formula of the food allows, supplementation with additives along the LAB strains could be a desired alternative, e.g., the addition of phenylalanine along with *L. reuteri* to food in order to increase PLA release.

For an advance in academic studies, enhancement in protection and the safety of products by LAB as probiotics and biopreservatives could be pursued. For food industrial researchers as well, the isolation, formulation, and industrialization of LAB antifungal bioactive metabolites could be of interest.

## Figures and Tables

**Table 1:** The most common synthetic antifungal preservatives, their negative effect on human health, and fungi that have developed partial resistance to them.

Preservatives	Food	Health Effects	Resistant Fungi	References
Benzoate	Fruit products Acidic foods Margarine Cereals Meat Carbonated drinks	Neurotransmission and cognitive functioning Hyperactivity and allergic reactions Genotoxic Clastogenic intercalation in the DNA structure		[19] [22] [23]
			*Zygosaccharomyces bailii*	[24]
			*Aspergillus flavus*	[25]
			*Aspergillus niger* and *Penicillium notatum*	[5]
			*Elymus repens* and *A. niger*	[26]
			*Aspergillus conicus*, *Penicillium*, *Cladosporium* and *Wallemia*	[27]
Propionate	Breads and other baked goods	Hypersensitivity Visual irritability Restlessness Inattention Sleep disturbance		[19]
			*E. repens* and *A. niger*	[26]
			*A. conicus*, *Penicillium*, *Cladosporium* and *Wallemia*	[27]
			*Penicillium expansum* and *Penicillium roqueforti*	[28]
			*P. roqueforti*	[29]
Sorbate	Syrups Dairy products Cakes Mayonnaise Margarine Processed meats	Cytotoxic and genotoxic effects DNA breakage Irritant to respiratory epithelium	*P. roquefortii*	[19] [30] [31] [32]
			*A. flavus*	[32]
			*P. notatum* and *A. niger*	[5]
			*Rhizopus nigricans*	[4]
			*E. repens* and *A. niger*	[26]
			*A. conicus*, *Penicillium*, *Cladosporium* and *Wallemia*	[27]

**Table 2:** Selected LAB studied for their antifungal metabolites, fungal spectrum of activity, and their in situ application in the last 10 years.

LAB Isolate	Antifungal Compound	Activity Spectrum	Food Product	Reference
*L. pentosus* G004 *L. fermentum* Te007 *L. paracasi* D5 *Pediococcus* *pentosaceus* Te010	Protein-like compounds	*A. niger* and *Aspergillus oryzae*	Bread Tomato Cheese	[81]
*L. amylovorus* DSM 19280	Acetic acid Lactic acid Hydrocinnamic acid Azelaic acid 4-Hydroxybenzoic acid	*P. expansum*	Cheddar cheese	[62]
*L. plantarum* LR/14	Antimicrobial peptides AMPs LR14	*A. niger*, *Rhizopus stolonifera*, *M. racemosus* and *P. chrysogenum*	Wheat grain	[82]
*L. plantarum*	Phenolic acids Organic acids	*F. culmorum* *P. expansum* MUCL2919240	Barley malt Bread grapes	[75] [83]
*L. fermentum*, *L. plantarum*	Phenyllactic acid Organic acids	*A. flavus*, *Penicillium citrinum*, *Penicillium griseufulvum*, *A. niger* and *A. fumigatus*	Cocoa beans	[76]
*L. reuteri*	Reuterin	*P. chrysogenum* and *M. racemosus*	Yogurt	[84]
*L. pentosus*, *L. plantarum*, *L. brevis*, *Lactobacillus delbrueckii*, *L. fermentum*, *Lactococcus lactis* and *Lc. mesenteroides*	Hydrogen sulphide and lactic acid	*Penicillium oxalicum*, *Fusarium verticillioides* and *A. niger*	Fruits and vegetables	[57]
*Lactobaciullus* strains	Organic acids	*P. chrysogenum* and *A. favus*	Caciotta cheese	[85]
*L. plantarum* CECT 749	Gallic, chlorogenic, caffeic and syringic acids	*Fusarium* spp. *Penicillium* spp. and *Aspergillus* spp.	Bread	[86]
Number of LAB strains isolated from Kimchi	Lactic acid and acetic acid	*Cladosporium* sp. YS1, *Neurospora* sp. YS3, and *P. crustosum* YS2	Rice cake	[59]
*Leuconostoc* spp. *L.* *reuteri* and *L.* *buchneri*	Organic acids such as lactic acid, acetic acid and propionic acid	*Aspergillus*, *Eurotium*, *Penicillium*, *Cladosporium* and *Wallemia* spp.	Milk bread rolls	[26]
*L. plantarum* CH1, *L. paracasei* B20 and *Lc. mesenteroides* L1	Lactic acid and acetic acid	*M. racemosus*, *Penicillium commune*, *Yarrowia lipolytica*, *A. tubingensis*, *A. flavus* and *Paecilomyces*	Sour cream and sourdough bread	[66]
